# Challenges of Diagnosing Mendelian Susceptibility to Mycobacterial Diseases in South Africa

**DOI:** 10.3390/ijms241512119

**Published:** 2023-07-28

**Authors:** Denise Scholtz, Tracey Jooste, Marlo Möller, Ansia van Coller, Craig Kinnear, Brigitte Glanzmann

**Affiliations:** 1 DSI-NRF Centre of Excellence for Biomedical Tuberculosis Research, South African Medical Research Council Centre for Tuberculosis Research, Division of Molecular Biology and Human Genetics, Faculty of Medicine and Health Sciences, Stellenbosch University, Cape Town 7505, South Africa; dscholtz28@gmail.com (D.S.); tjooste@sun.ac.za (T.J.); marlom@sun.ac.za (M.M.); gkin@sun.ac.za (C.K.); 2 Centre for Bioinformatics and Computational Biology, Stellenbosch University, Stellenbosch 7600, South Africa; 3 South African Medical Research Council (SAMRC) Genomics Platform, Cape Town 7505, South Africa; ansia.vancoller@mrc.ac.za

**Keywords:** inborn errors of immunity, tuberculosis, genes, MSMD, transcriptomics, NGS

## Abstract

Inborn errors of immunity (IEI) are genetic disorders with extensive clinical presentations. They can range from increased susceptibility to infections to significant immune dysregulation that results in immune impairment. While IEI cases are individually rare, they collectively represent a significant burden of disease, especially in developing countries such as South Africa, where infectious diseases like tuberculosis (TB) are endemic. This is particularly alarming considering that certain high penetrance mutations that cause IEI, such as Mendelian Susceptibility to Mycobacterial Disease (MSMD), put individuals at higher risk for developing TB and other mycobacterial diseases. MSMD patients in South Africa often present with different clinical phenotypes than those from the developed world, therefore complicating the identification of disease-associated variants in this setting with a high burden of infectious diseases. The lack of available data, limited resources, as well as variability in clinical phenotype are the reasons many MSMD cases remain undetected or misdiagnosed. This article highlights the challenges in diagnosing MSMD in South Africa and proposes the use of transcriptomic analysis as a means of potentially identifying dysregulated pathways in affected African populations.

## 1. Introduction

Inborn errors of immunity (IEI) are genetic disorders caused by germline mutations, leading to functional defects in the proteins of the immune system [1,2]. These disorders present with increased vulnerability to infections or significant immune dysregulation, which results in immune impairment with a broad range of clinical manifestations [1,3].

Individuals suffering from IEI are not only predisposed to recurrent and chronic infections—despite completion of appropriate treatment clearing previous infections—but also have an increased risk of developing autoinflammatory, autoimmune, and allergic responses, significantly increasing their morbidity and mortality [4,5,6,7]. Individuals with IEI allow for the unique opportunity to study and understand the function of affected pathways in the human immune system [5].

**Figure 1:**
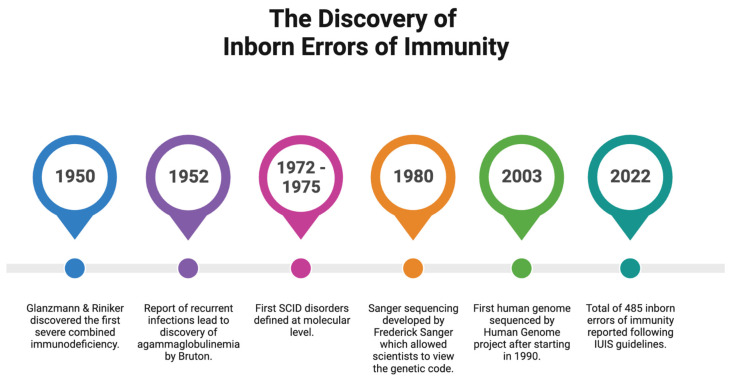
The history of the discovery and diagnosis of inborn errors of immunity. Created with BioRender.com (accessed on 25 June 2023). Abbreviations: SCID, severe combined immunodeficiency.

**Figure 2:**
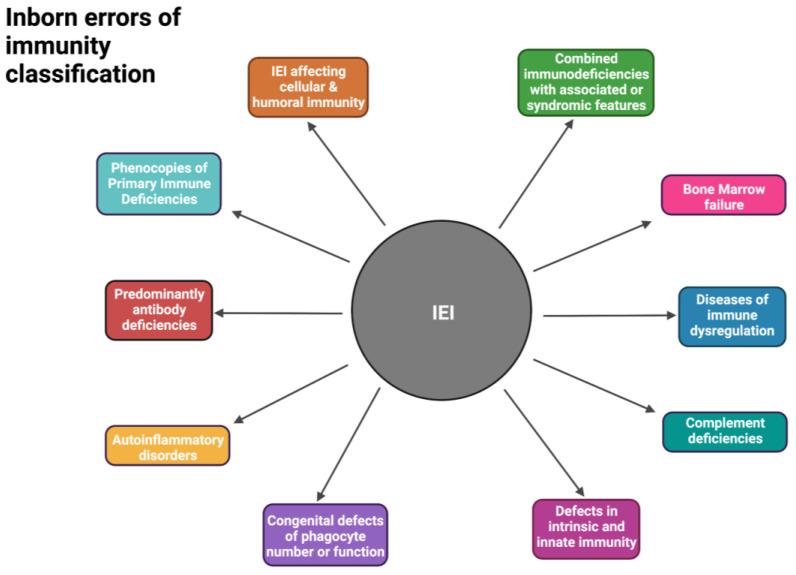
Broad classification of inborn errors of immunity according to the International Union of Immunological Societies (IUIS) guidelines [9]. Created with BioRender.com (accessed on 25 June 2023).

The history of IEI dates back to 1950 when Glanzmann and Riniker reported an infant with severe lymphopenia and atrophy of the lymphoid tissues who died due to infections early in life, followed by a multitude of important milestones that led us to where we are with the IEI diagnosis today (Figure 1) [8]. Since 1970, the International Union of Immunological Societies (IUIS) has created an international expert committee that has provided both the clinical and research communities with updated genetic causes of immune deficiency and dysregulation. This committee applies rigorous criteria to classify gene defects as novel causes of IEI (Figure 2) [9,10].

There are three main criteria to consider novel gene defects; the first is that the candidate genotype of the patient is monogenic in nature and does not occur in individuals that do not present with the clinical phenotype while recognising that some conditions have incomplete penetrance. Additionally, experimental studies should be conducted to establish that the patient’s variant will alter the gene in such a way that it changes the gene product. Lastly, a causal relationship between the candidate genotype and the clinical phenotype must be confirmed by a relevant cellular phenotype, including, if possible, the rescue of a functional defect [9,10]. In light of this, multiple diagnostic gene panels are currently available to screen for known IEI-associated genes. These panels include between 200 and 407 genes, allowing for the molecular diagnosis of patients to be much more efficient and cost-effective than next-generation sequencing (NGS) technologies [5].

Understanding the influence of the host’s genetics on infectious disease susceptibility not only provides crucial insights into disease pathogenesis but also enables the discovery of potential drug targets and the implementation of more effective therapeutic strategies [5,11]. In addition to improving molecular diagnosis and therapies of specific IEI, the knowledge gained from investigating IEI can also be applied to other diseases for which similar underlying pathways are involved [5,10]. Thus far, genetic abnormalities in approximately 490 genes have been identified and described to cause 485 different IEI phenotypes [5,9,12]. These highly heterogeneous IEI phenotypes include antibody deficiency, T- and B-lymphocyte deficiency, complement deficiency, autoimmunity, lymphoproliferative syndromes, and immune dysregulation (Figure 2) [5,9].

## 2. Inborn Errors of Immunity in South Africa

While individual IEI cases are rare, collectively they constitute a significant burden of disease [13,14]. Inborn errors of immunity were initially thought to affect approximately 1 in 10,000 to 1 in 50,000 births but with the improvements in clinical diagnosis, it was discovered that the actual number is likely around 1 in 1000 to 1 in 5000 births [1,15].

It is speculated that the prevalence of IEI should be similar in South Africa to other, well-resourced countries, which varies between 5.38/100,000 in France and 10.3/100,000 in a Mayo Clinic epidemiologic study, and 86.3/100,000 in a telephone survey executed by Boyle and Buckley in the USA [2,16,17,18]. If that is true, then the number of patients diagnosed with IEI, based on the mid-2017 population of 56.52 million, should be between 3040 and 48,755 [2,19].

Despite a stable increase in the diagnosis of IEI in Africa, the IEI burden remains severely underestimated in low- to middle-income countries, with less than 500 total cases reported from South Africa in 2020 specifically [20,21]. While this may be partly due to a lack of early childhood diagnosis or delayed onset of disease, it can largely be attributed to challenges associated with awareness, resources, as well as patient care and registration to national primary immunodeficiency registries [6,9,14].

According to a 2020 global systematic review [13], only 168 patients were included in the South African National Primary Immunodeficiencies Registry (PINSA), of which the last paper was published in 2014 [2,22]; however, the most recent numbers include 437 patients on the South African registry (PINSA, unpublished). The absence of more comprehensive IEI databases significantly impacts our ability to diagnose affected individuals and improve patient care and outcome.

Additionally, the high burden of more confounding/urgent healthcare problems decreases the available resources to diagnose IEI [20]. South Africa’s underlying burden of endemic TB is one of the largest in the world, with more than 500 cases per 100,000 population [23]. The heterogeneity and varied phenotypes within specific IEI [24,25,26], given the range of genetic polymorphisms in South African populations, further complicate accurate early diagnosis [20]. The diversity and unique genomic traits of South Africans have phenotypic implications arising from unique genetic factors affecting both simple and complex phenotypes, such as altered disease susceptibility [27,28,29].

Lastly, the lack of resources and consistent diagnostic and treatment facilities on the continent also critically affects patients [20]. Genetic services remain scarce in low- and middle-income countries such as South Africa [30]. Without an accurate molecular diagnosis, clinicians are not able to understand the disease phenotype well, resulting in a lack of precise treatment for patients and results in a high overall mortality rate in South Africans with IEI, ranging from 25 to 34.5% [20,30,31]. In cases with confirmed diagnoses, treatment regimens are able to be altered by, for instance, increasing clinical surveillance, extending treatment duration, as well as potential supplementation with IFN-γ, to name a few [32].

### 2.1. Susceptibility to Mycobacterial Disease

Certain high penetrance mutations in the immune system that cause IEI such as Mendelian Susceptibility to Mycobacterial Diseases (MSMD), common variable immunodeficiency (CVID), and chronic granulomatous disease may put individuals at higher risk for mycobacterial diseases, including TB, or a wide range of fungal, viral, and bacterial infections [2,12,14,33,34]. Approximately 5% of infected, immunocompetent individuals will develop clinical TB within two years of infection and may exclude the latency phase (approximately 95% of infected individuals), which is characterized by a positive tuberculin skin test (TST) and/or interferon-gamma (IFN-γ) release assay (IGRA) in the absence of clinical symptoms [14,35,36]. This is known as ‘primary’ TB and is most commonly seen in children [37]. The higher rates of active TB outcomes in children than expected may suggest a genetic basis for the increased risk of developing active TB, in addition to environmental factors [14,38,39].

Since resistance to mycobacterial infection relies on an efficacious immune system and the immune response is largely determined by the host’s genetic makeup, IEI cases that relate to mycobacterial infection are of particular concern in TB-endemic areas like South Africa. MSMD and other IEI cases associated with TB susceptibility could be more prevalent than believed but may be either under- or misdiagnosed [14,32].

#### 2.1.1. Age/Sex Influence (the Wonder Years)

**Figure 3:**
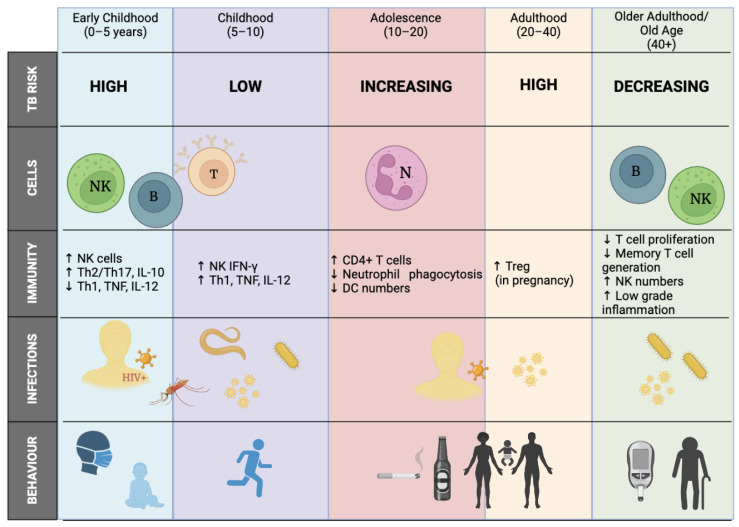
Tuberculosis risk changes with age and is influenced by hormones, changes in immune cell activity, behaviours, and environmental factors. Co-infections include HIV risk at birth, malaria, helminths, influenza, etc., in childhood and all throughout the life stages. Behaviours that influence risk cover infant risk to adult TB, exposure to the outdoors, and includes drinking, sexual activity, and smoking risk from adolescence to diabetes and old age later in life. Adapted from [40]. Abbreviations: B, B cells; DC, dendritic cells; IFN, interferon; MN, monocytes; N, neutrophils; NK, natural killer cells; T, T lymphocytes; Th, T helper; Treg, regulatory T cells; WBC, white blood cells; TB, tuberculosis; HIV, human immunodeficiency virus, ↑, increase; ↓, decrease. Created with BioRender.com (accessed on 25 June 2023).

In TB-endemic settings, the risk of infection with *Mycobacterium tuberculosis* (*M. tb*) increases throughout childhood due to cumulative exposure (Figure 3) [40]. Children older than 5 years and adolescents (10–15 years) have lower TB incidence rates compared to very young children and older adults [41,42,43]. This is often referred to as the “golden age” or “wonder years” and these children may be immunologically protected against developing active TB disease [40,41,42]. Disease phenotype also differs by age, where young children generally have disseminated disease and adolescents develop adult-type pulmonary disease. Differences in phenotype also exist between sexes, with adolescent girls generally having a higher TB risk and earlier progression to disease until early adulthood in comparison to adolescent boys [40]. Figure 3 illustrates these changes in TB risk over time. In South Africa, however, severe, persistent, unusual, and recurrent (SPUR) TB has been investigated in a paediatric population (ages 5–15), who are normally immunologically protected. Understanding why only a subset of infected children in their “golden age” develop SPUR TB in the absence of secondary immunodeficiencies such as HIV, remains to be fully elucidated.

#### 2.1.2. BCG-Vaccine Strategy in TB-Endemic Countries

The Bacillus Calmette–Guerin (BCG) vaccination is given at birth for the prevention of paediatric TB, particularly in TB-endemic countries [44]. BCG is hypothesised to train the innate immune system to offer protection from respiratory tract infections. It induces a protective efficacy against tuberculous, meningitis, and miliary disease of more than 70% on average in HIV-unaffected children [26,44]. The BCG vaccination was introduced in South Africa during the 1950s and has been routinely administered to all children at birth since 1973 [44,45,46].

The BCG vaccination is generally safe in immunocompetent children; however, because BCG is a live vaccine, it may result in adverse effects in immunocompromised children. Although rare, when BCG dissemination is recorded in HIV-uninfected children, it may indicate that an IEI is the cause of the severe effects [7,44]. For instance, in severe combined immunodeficiency (SCID) patients, these BCG complications were defined by clinical, microbiological, and/or histopathology findings and were classified as localized or disseminated TB [47].

Previous studies have indicated that while the BCG vaccine is not entirely effective at preventing infection, it is still routinely used to prevent severe and disseminated TB in children [7]; however, it has been found that the vaccine had no protective effect in children with IEI and has also been found to cause disseminated mycobacterial disease in some children with IEI related to susceptibility to mycobacterial diseases [7,45,46].

## 3. Mendelian Susceptibility to Mycobacterial Diseases

Although IEI patients are known to be susceptible to a multitude of infections and autoimmunity manifestations, some seem to display a specific predisposition to mycobacterial infections, such as MSMD [48,49]. MSMD is a term to describe a group of IEI that result in selective susceptibility to weakly virulent pathogenic mycobacteria such as the BCG vaccine and environmental mycobacteria [32,48]. Individuals presenting with MSMD are at higher risk of being infected with the *M. tb* complex (MTBC), which comprises a significantly genetically similar group of bacteria that cause tuberculosis [32].

### 3.1. Genes Involved in MSMD

**Figure 4:**
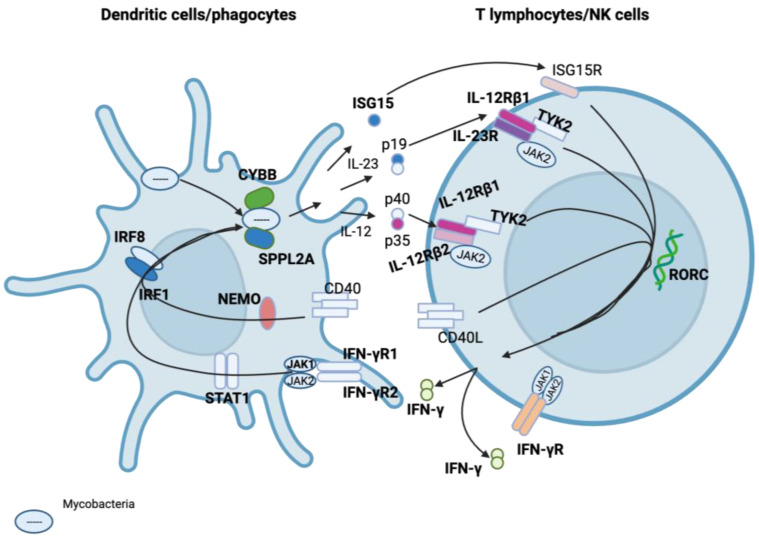
Inborn errors of the IFN-y-IL-12 pathway in patients with conditions grouped under MSMDs. Some of the genes that have been described and are associated with MSMD are shown in bold. Arrows indicate interactions between genes in the pathway. Adapted from [7]. Abbreviations: IRF8 Interferon regulatory factor 8; IRF1, Interferon regulatory factor 8; NEMO, Inhibitor of Kappa beta Kinase γ; STAT1, signal transducer and activator of transcription 1; SPPL2A, signal peptide peptidase-like 2A; CYBB, cytochrome b-245 beta chain; JAK1, Janus kinase 1; JAK2, Janus kinase 2; CD40, cluster of differentiation 40; IFN-γR1, Interferon gamma receptor 1; IFN-γR2, Interferon gamma receptor 2; IL-12, interleukin 12; IL-23, interleukin 23; ISG15, interferon-stimulated gene 15; p19, 19 kDa protein; p40, 40 kDa protein; p35, 35 kDa protein; IL-12Rβ1, interleukin 12 receptor subunit beta 1; IL-12Rβ2, interleukin 12 receptor subunit beta 2; IFN-γ, interferon gamma; CD40L, cluster of differentiation 40 ligand; TYK2, tyrosine kinase 2; ISG15R, interferon-stimulated gene 15 receptor; RORC, RAR-related orphan receptor gamma; IL-23R, interleukin 12 receptor. Created with BioRender.com (accessed on 25 June 2023).

To date, 19 genes have been associated with conditions grouped under MSMD [32,48]. These include interleukin 12 receptor subunit beta 1 (*IL12RB1*), interleukin 12 receptor subunit beta 2 (*IL12RB2*), subunit beta of interleukin 12 (*IL12B*), interferon gamma receptor 1 (*IFNGR1*), and interferon gamma receptor 2 (*IFNGR2*) and have been described as genetic defects generally associated with a disruption in Interferon-gamma (IFN-γ) and the Interleukin-12 (IL-12) pathway. These may affect the interactions of mononuclear phagocytes and T helper cells surrounding the synthesis and response to IFN-γ, which is essential for the clearance of mycobacterial infections (Figure 4) [7,12,48,50,51]. The allelic heterogeneity of the associated genes results in at least 34 well-defined genetic disorders, with varying modes of inheritance (autosomal recessive, autosomal dominant, or X-linked) and clinical phenotypes [51,52,53,54].

Although MSMD has inflated levels of allelic similarity, it displays physiological similarity, since all the associated gene defects eventually impair IFN-γ-mediated immunity. The defects that occur can either obstruct the production of IFN-γ or cause an abnormal response to IFN-γ signalling (Table 1) [48,55].

The clinical manifestations of patients have been associated with the specific gene/s of interest and include the correlation of pathogens to the genetic cause of disease [48]. Patients presenting with defects in IFN-γ production originating from mutations in *IL12RB1* and *IL12B* generally suffer from disease caused by Salmonella infection, as well as Candida to a lesser extent. In the matter of IFN-γ response defects, the presence of multifocal osteomyelitis could indicate a partial autosomal dominant (AD) IFN-γR1, or partial autosomal recessive (AR) or partial AD *STAT1*, which results in loss of function in these genes [48,57]; additionally, patients presenting with an eradicated IFN-γ response, thus with a complete deficiency in IFN-γR1 and IFN-γR2, are more prone to viral diseases such as cytomegalovirus [57,58].

### 3.2. Clinical Features and Diagnosis

The diagnosis of MSMD requires meticulous clinical evaluations, including functional immunophenotyping and a molecular diagnosis where possible [32,54,59]. The clinical presentation of MSMD is extensive—ranging from less severe forms that have a later onset in life to early-onset, severe, disseminated, and persistent life-threatening mycobacterial disease [32,48,54,59].

The findings on standard clinical workups are often normal and not suggestive of an IEI such as MSMD; therefore, clinical suspicion is very important [8,21,26]. In addition, IEI cases such as severe combined immunodeficiency, common variable immunodeficiency, combined immunodeficiency, chronic granulomatous disease, and hyper-IgM syndromes, are essential to rule out in the process of diagnosing MSMD patients [32]. Clinical examinations include the analysis of medical and family histories, vaccination history, and physical examinations; additionally, routine laboratory testing, such as HIV tests, conventional TB tests, Qiagen QuantiFERON TB IFN-γ release assay, etc., is included to exclude other illnesses or causes of immunodeficiency, e.g., interferon axis deficiencies [7]. In a South African cohort, some cases presented with more virulent mycobacteria such as *M. tb*, whereas others presented with SPUR *M. tb* infections, which could be indicative of MSMD or other mycobacteria-related IEI [2,7,32]. Within the SPUR TB clinical phenotype, persistent infections were defined as infections that required a longer duration of therapy than normal, or where the patients had a lack of response to the appropriate regimen for more than two months [7]. It is referred to as unusual when infections occurred at unexpected sites, such as dissemination of TB to the ears or infection with an unusual organism, which only causes disease in compromised immune systems [7]. Additionally, these patients had TB recurrence, defined as two or more occurrences at least one year apart, despite the completion of therapy and elimination of the previous infection [7]. A history of previous infant death in the family due to infections or a known family history of IEI are also red flags [2]. Furthermore, infection with unusual mycobacterial isolates with atypical presentations, such as BCG/non-tuberculous mycobacteria (NTM) was present and includes infection with MTBC. MTBC was predominant in the South African MSMD cohort [32]. Interestingly, this is in contrast to the developed-world, which could suggest that MSMD clinical definitions should be adapted in TB-endemic settings and would need to include the relevance of BCG dissemination as a marker, recurrent meningococcal infections, and infections with atypical mycobacteria [2,32]. For healthcare practitioners, the use of gene panels with known genes is the most cost-effective option to diagnose MSMD [57].

#### 3.2.1. Consanguinity

Several studies have noted that the prevalence of IEI is closely related to consanguineous marriages [51,60]. In populations with high rates of consanguinity, such as East Asian, North African, and Middle Eastern countries, MSMD was reported to be more frequent in childhood mycobacterial disease cases than in developed countries [51,60]. This resulted in MSMD initially being categorized as autosomal recessive due to the high frequency of multiple-case siblings, as well as consanguineous relatives [51]; however, contrary to North Africa and other countries with high rates of consanguinity (>60%), a study investigating an MSMD cohort in South Africa found that consanguinity was rare and occurred in less than 2% of patients [19,20].

#### 3.2.2. Effectiveness of BCG Vaccinations

With the lack of BCG dissemination and insufficient prevention of childhood TB in a South African MSMD cohort, there is uncertainty regarding the effectiveness of the vaccine in the African context [61,62]. The licensed BCG vaccine formulations have not been systematically compared in regard to their viability and immunogenicity, and of the controlled clinical trials evaluating these aspects, none were carried out in Africa [61,62]. The immunogenicity of vaccines can vary notably between formulations and individuals, and individual responses can differ within ethnic groups from the same location. This indicates varied vaccine responses and antibody decline rates, which suggests a possible genetic influence [61,63]. Jethwa et al. studied ethnic diversity and immunogenicity in COVID-19 vaccine trials and found that there is evidence to suggest that COVID-19 affects different ancestries disproportionately [63]. Due to this, clinical vaccine trials should assess vaccine efficacy and safety using a diverse mix of ethnicities, especially for vaccines against diseases with high prevalence or endemicity in admixed populations. The insufficiency of ancestral diversity in vaccine trials means efficacy may differ between countries and may result in health disparities [63]. It is thus possible that these disparities in COVID-19 vaccine efficacy may be applicable to BCG vaccinations as well.

#### 3.2.3. Inheritance of Maternal Immunity

In addition to our hypothesis that the BCG vaccinations may not illicit a proper immune response in the cohort of interest due to the lack of BCG dissemination in suspected MSMD cases, the potential that maternal immunity might affect an infant’s response to vaccination should also be considered. Infants are born with immature immune systems, making them more susceptible to infectious pathogens as they are not able to effectively respond; however, during pregnancy, maternal antibodies are actively transported to the newborn during the first weeks to months of life [64]. Maternal vaccination may also impact the transfer of immune cells to their offspring, as breastfeeding children enables the transfer of vaccine-specific maternal antibodies through breastmilk [64,65]; however, it has been repeatedly found that maternal antibodies can inhibit the immune response of children to vaccines and may thus affect their efficacy [64]. Its effect on the effectiveness of BCG vaccinations in children and thus BCG dissemination in MSMD cases remains unknown.

#### 3.2.4. Challenges of Diagnosing MSMD in an Endemic-TB Setting

Despite the notable scientific progress made to date, there are still many patients with clinical presentations resembling MSMD for whom no molecular cause has been found, suggesting the need for further unbiased investigative approaches. It is therefore crucial to identify region-specific risk factors and to establish awareness of increased TB risk in the early diagnosis of children with suspected or confirmed IEI [32]. Furthermore, in TB-endemic regions, there is a demand for appropriate clinical algorithms to identify at-risk patients and a diagnostic approach for IEI in general, as well as suspected MSMD patients specifically. A South African team investigated the use of SPUR TB as a marker for MSMD, considering the endemicity of TB [32]. Using the previously mentioned criteria, a molecular diagnosis using WES identified potential candidate variants in genes that have previously been associated with MSMD [32,33]. Only 41% of these variants were classified as pathogenic, no candidate genes were identified in 9%, and VUS were found in 50% of cases. The sensitivity of using SPUR TB as a clinical definition for identifying MSMD molecularly was 78%; in addition, the accuracy of MSMD diagnosis was 35% [32]. The use of molecular testing is crucial to determine and diagnose the immune deficit, as it can impact clinical outcomes and the response to specific therapies [32]. In South Africa, molecular diagnosis via WES or WGS is generally only available in a research capacity, due to limited funding for genomic medicine [32]. Among Africans, the impact of delayed diagnosis has resulted in an overall mortality rate for patients with IEI of approximately 25–34.5% [20]. A South African registry-based survey was able to evaluate that 32% of diagnosed patients were reported to have accessed intravenous immunoglobulin treatment, only 4% had access to subcutaneous immunoglobulin, and only six out of eleven SCID patients were able to receive stem cell transplants [66]. The adaptation of diagnostic criteria for diagnosing MSMD in South Africa should thus be prioritised in alignment with international guidelines to ensure prompt diagnosis and treatment of patients. A paper by Cornelissen et al. has put forward recommendations to improve the diagnostic yield of patients with underlying MSMD, which includes the elimination of secondary immunodeficiencies in the context of SPUR TB. Furthermore, positive cultures for BCG or NTM in the absence of HIV or any secondary causes should be brought to the attention of clinicians by the microbiology laboratories to investigate further [32].

### 3.3. Genetic Approaches to Molecular Diagnosis

To receive a full diagnosis, functional tests and genetic studies are needed. There is a wide variety of functional tests available for MSMD diagnosis, such as ELISA for IFN-γ and IL-12, STAT1 phosphorylation, and the detection of anti-IFN-γ autoantibodies [48,57,67]; however, the use of these techniques requires qualified staff, making the full diagnosis of MSMD only possible in specialized immunology laboratories. Using next-generation sequencing (NGS) techniques to confirm mutations could overcome some of these limitations in functional testing, including being less time-consuming and more cost-effective. Nonetheless, genetic results still need functional validation of the identified mutations [57]. Genomic testing has become the gold standard for molecular diagnosis in IEI cases, as the application of WES, a popular NGS tool, has confirmed a molecular diagnosis in the range of 30–40% in IEI [32].

#### 3.3.1. NGS and Variants of Unknown Significance (VUS)

A large proportion of patients with clinically suspected MSMD do not have mutations in the known disease-causing genes available on commercial gene panels [51,57]. In these situations, the use of NGS techniques, such as whole exome sequencing (WES) and whole genome sequencing (WGS), may be required. Next-generation sequencing has allowed the augmented identification of novel IEI due to mutations in genes that encode for the immune response [5]. The use of WES and WGS as universal molecular gene testing methods may improve the diagnosis of IEI [57]; however, evidence from the literature has suggested that this may not be adequate, as WES and WGS have only been able to produce a concrete diagnosis in 25–60% of cases, leaving a proportion of patients without diagnosis [68,69]. Additionally, according to a 2018 meta-analysis, in Mendelian conditions such as MSMD, the failure to identify a conclusive molecular cause occurs in about 70–75% of conditions [68,70].

A frequently used approach for the diagnosis of IEI is to start with an NGS tool—either gene panels or WES/WGS, to identify potential mutations and conclude with functional testing to confirm the mutations found; however, the large number of phenotypes associated with specific diseases and the identification of gene variants of unknown significance following WES/WGS further complicates accurate IEI diagnosis [33].

In a South African MSMD cohort studied by Cornelissen et al., it was discovered that in 9% of cases, no candidate genes were identified; however, the identification of a candidate gene of unknown significance occurred in 50% of cases [32]. Functional immunophenotyping of the IFN-γ-IL-12 was performed for all patients, of which all pathogenic variants found showed an impaired response in the pathway [7,32]. In the patients with variants of unknown significance (VUS) or no significant variant, impaired functioning of the pathway was also found, such as in a patient with a VUS in IL12RB2. The aforementioned variant fits the associated phenotype of the patient but requires further investigation and functional validation of its involvement [7,32]. Despite needing functional phenotyping to confirm VUS as a pathogenic variant, these studies found the impairment of the IFN-γ-IL-12 pathway in combination with SPUR infections as highly suggestive of MSMD [7,32].

#### 3.3.2. Under-Representation in the Human Reference Genome

Even with the rise in the use of human genome sequencing to identify millions of previously unknown genetic variants, it remains unable to fully elucidate pathogenic variants in African populations. This might be due to the under-representation of African populations in sequencing efforts, even though reports stipulate that Africans have the most diverse population genetics [71,72]. While almost a seventh of the world population is comprised of Africans, genomic research has been biased towards Eurasian populations with very few studies conducted on individuals of African ancestries [28,71,73]. Despite the fact that the 1000 Genomes Project has exponentially increased our understanding of genetic variation worldwide, it still lacks sufficient representation of African, and more specifically, Southern African populations [74]. Projects such as the African Genome Variation Project, have aimed to mitigate this problem by providing a resource with dense genotypes from 1481 individuals and whole-genome sequences from 320 individuals across sub-Saharan Africa [75].

This lack of representation further extends to the human reference genome, from which the identification of novel or known genetic variants from NGS data is largely dependent [76]. The underrepresentation of African populations may thus exclude them from understanding disease aetiology as well as the detection and diagnosis of disease, as seen in MSMD [77]. One of the many implications of excluding African populations is the efficacy of medications—cures that are effective in certain populations are ineffectual in others [45]; therefore, additional sequencing efforts from diverse African populations are required to contribute to large-scale publicly available datasets and facilitate the construction of African-specific reference genomes in order better characterise the spectrum of variation in humans [71,74,76]. African genetic diversity may give insight to elucidate novel disease susceptibility, which increases the possibility of correct diagnosis, with the significant potential to inform clinical care [73]. The lack of ancestry-specific genomes has thus resulted in healthcare inequalities and hinders the implementation of precision medicine in people of African ancestry [71,75].

#### 3.3.3. Lack of Transcriptomics as a Diagnostic Tool

The identification of definitive disease-causing mutations may be confounded by the expression levels that are modulated by mutations occurring in non-coding regions and in genes that are not currently linked to the disease or phenotype, such as VUS [68]. Expression quantitative loci (eQTL) is a locus that explains a fraction of the genetic variance of a gene, and these eQTLs elicit a powerful effect on the expression of a large number of genes [68,78,79]. Single-nucleotide polymorphisms (SNPs) on eQTLs can affect the transcriptional levels of other RNAs, in turn, modifying protein expression and causing phenotypic changes to cells in some immunological cases [68,79]. It has been shown that these eQTLs have more pronounced effects than sex and age on immune regulation, proving the importance of transcriptomics in some cases [68,80]. This might offer new insights into the pathways involved in the susceptibility to mycobacterial infection, especially in African populations, in which various VUS have been found [81,82].

Von Both et al. attempted the exploration of further immunological consequences of the impairment of the IFN-γ/IL-12 pathway in MSMD by analysing the transcriptomic response of PBMCs. They found sixty-four genes that were significantly differentially under-expressed, which might be considered potential biomarkers of the interferon response, critical in the susceptibility to mycobacteria [81]. Additionally, a transcriptome dataset collection has been curated by Bougarn et al. in an effort to investigate the spectrum of inborn errors of immunity and the degree to which gene function is lost or altered [83]. Studies are thus investigating the value of adding transcriptomic profiling to existing genomic diagnostic approaches, such as WES/WGS, to help bridge the gap in what distinguishes the protective and susceptible response to infection [81,82]. The use of transcriptomic analysis might be pivotal in identifying dysregulated pathways or genes affected in African populations that have not been implicated in the largely European-based references.

## 4. Conclusions

South Africa is a genetically diverse country known to be burdened by infectious diseases such as TB and HIV, which amplify the challenge of diagnosing IEI. It is imperative to identify individuals with MSMD in these high-burden countries, as they could potentially acquire early and devastating TB infections. It is believed that IEI cases such as MSMD and others that are associated with TB susceptibility are being overshadowed by the HIV/TB co-epidemics and are more common than previously assumed. This promotes the idea that MSMD can be hidden under severe, persistent, unusual, and recurrent infections. Even more specifically so in the African continent, where a variety of endemic diseases and a lack of consistent diagnostic and treatment facilities are prevalent problems. Additionally, the extensive lack of medical resources to help diagnose these patients inhibit the potential capacity building within the clinical and medical research settings in South Africa.

From a South African perspective, patients present with a clinical phenotype in contrast with the developing world settings. This increases the difficulty in diagnosing patients with a clear IEI, as human references are largely based on European populations. Additionally, the increased TB incidence in a setting with an implemented BCG vaccine strategy, low incidence of consanguinity, and differences that cannot be explained by age or sex, reiterate the need for evaluating region-specific risk factors.

The diagnosis of MSMD remains difficult due to a lack of awareness, clinical challenges in identifying MSMD phenotypes, and trials in molecular diagnoses with NGS tools to identify appropriate candidate genes. These challenges are stratified by the intense task of filtering genes and interpreting them considering the patient’s clinical phenotype, especially in genes of unknown significance and those residing in the non-coding regions of the DNA. Further functional validation is needed to confirm the candidate genes and is currently only available in specialised research laboratories. The introduction of transcriptomic sequencing-based analysis for patients presenting with suspected MSMD, with no abnormal haematological profiling, may be used to provide unparalleled insights into the workings of the cell necessary to diagnose patients. 

## Figures and Tables

**Table 1:** Mendelian susceptibility to mycobacterial disease (MSMD)-associated genes and their mutation effects. Adapted from [48,51] and data from OMIM [56].

Gene	MSMD-Causing Mutation Effect
*IL12RB1*	Impaired *IFN-γ* production
*IL12RB2*	Impaired *IFN-γ* production
*IL12B*	Impaired *IFN-γ* production
*IFNGR1*	Impaired antimicrobial, antiviral, and anti-tumour responses, impaired cellular response to *IFN-γ*
*IFNGR2*	Impaired antimicrobial, antiviral, and anti-tumour responses, impaired cellular response to *IFN-γ*
*STAT1*	Defective type I and type II *IFN* responses
*CYBB*	Oxidative burst defects in macrophages
*IRF8*	Loss of myeloid dendritic cells
*TYK2*	Dysregulation of essential signalling for Type I and Type II cytokines
*ISG15*	Impaired IFN-γ production
*RORC*	Disruption of IL-27/IFN-γ immunity
*NEMO*	Impaired T-cell response and CD40-dependent production of *IL-12*
*SPPL2A*	Dysfunction of antigen-presenting cells
*JAK1*	Dysfunctional INF-α, IL-6/10, and IL-12 signalling
*IL23R*	Dysfunction in the receptor of Type I and Type II cytokines
*TBX21*	Th1 cell-specific transcription factor that functions as a regulator of IFN-γ expression
*ZNFX1*	Can initiate *IFN* expression following exposure to viruses
*PDCD1*	Immune-inhibitory receptor expressed in activated T cells
*IFNG*	Codes for *IFN*-γ used for communication between cells to trigger the protective defences of the immune system that help eradicate pathogens

Abbreviations: IRF8 Interferon regulatory factor 8; IRF1, Interferon regulatory factor 8; NEMO, Inhibitor of Kappa beta Kinase γ; STAT1, signal transducer and activator of transcription 1; SPPL2A, signal peptide peptidase-like 2A; CYBB, cytochrome b-245 beta chain; JAK1, Janus kinase 1; JAK2, Janus kinase 2; IFN-γR1, Interferon gamma receptor 1; IFN-γR2, Interferon gamma receptor 2; IL-12, interleukin 12; IL-23, interleukin 23; ISG15, interferon-stimulated gene 15; IL-12Rβ1, interleukin 12 receptor subunit beta 1; IL-12Rβ2, interleukin 12 receptor subunit beta 2; IFN-γ, interferon gamma; TYK2, tyrosine kinase 2; ISG15R, interferon-stimulated gene 15 receptor; RORC, RAR-related orphan receptor gamma; IL-23R, interleukin 12 receptor; TBX21, T-box transcription factor 21; ZNFX1, Zinc finger NFX1-type containing 1; PDCD1, programmed cell death protein 1.

## Data Availability

Not applicable.

## References

[1] Tangye S.G., Al-Herz W., Bousfiha A., Chatila T., Cunningham-Rundles C., Etzioni A., Franco J.L., Holland S.M., Klein C., Morio T. (2020). Human Inborn Errors of Immunity: 2019 Update on the Classification from the International Union of Immunological Societies Expert Committee. J. Clin. Immunol..

[2] Eley B., Esser M. (2014). Investigation and management of primary immunodeficiency in South African children. S. Afr. Med. J..

[3] Delmonte O.M., Castagnoli R., Calzoni E., Notarangelo L.D. (2019). Inborn Errors of Immunity with Immune Dysregulation: From Bench to Bedside. Front. Pediatr..

[4] Bigley T.M., Cooper M.A. (2021). Monogenic autoimmunity and infectious diseases: The double-edged sword of immune dysregulation. Curr. Opin. Immunol..

[5] Rey-Jurado E., Poli M.C. (2021). Functional genetics in inborn errors of immunity. Future Rare Dis..

[6] Staels F., Collignon T., Betrains A., Gerbaux M., Willemsen M., Humblet-Baron S., Liston A., Vanderschueren S., Schrijvers R. (2021). Monogenic Adult-Onset Inborn Errors of Immunity. Front. Immunol..

[7] Van Coller A., Glanzmann B., Cornelissen H., Möller M., Kinnear C., Esser M., Glashoff R. (2021). Phenotypic and immune functional profiling of patients with suspected Mendelian Susceptibility to Mycobacterial Disease in South Africa. BMC Immunol..

[8] Glanzmann E., Riniker P. (1950). Essential lymphocytophthisis, new clinical aspect of infant pathology. Ann. Paediatr..

[9] Tangye S.G., Al-Herz W., Bousfiha A., Cunningham-Rundles C., Franco J.L., Holland S.M., Klein C., Morio T., Oksenhendler E., Picard C. (2022). Human Inborn Errors of Immunity: 2022 Update on the Classification from the International Union of Immunological Societies Expert Committee. J. Clin. Immunol..

[10] Casanova J.-L., Conley M.E., Seligman S.J., Abel L., Notarangelo L.D. (2014). Guidelines for genetic studies in single patients: Lessons from primary immunodeficiencies. J. Exp. Med..

[11] Kwok A.J., Mentzer A., Knight J.C. (2021). Host genetics and infectious disease: New tools, insights and translational opportunities. Nat. Rev. Genet..

[12] Boisson-Dupuis S., Bustamante J. (2021). Mycobacterial diseases in patients with inborn errors of immunity. Curr. Opin. Immunol..

[13] Erjaee A., Bagherpour M., Van Rooyen C., van den Berg S., Kinnear C.J., Green R.J., Pepper M.S. (2019). Primary immunodeficiency in Africa—A review. South Afr. Med. J..

[14] Glanzmann B., Uren C., de Villiers N., van Coller A., Glashoff R.H., Urban M., Hoal E.G., Esser M.M., Möller M., Kinnear C.J. (2019). Primary immunodeficiency diseases in a tuberculosis endemic region: Challenges and opportunities. Genes Immun..

[15] Prokofjeva T., Lucane Z., Kovalova Z., Kurjane N. (2022). Inborn Errors of Immunity in Latvia: Analysis of Data from 1994 to 2020. J. Clin. Immunol..

[16] Bousfiha A.A., Jeddane L., Ailal F., Benhsaien I., Mahlaoui N., Casanova J.-L., Abel L. (2013). Primary Immunodeficiency Diseases Worldwide: More Common than Generally Thought. J. Clin. Immunol..

[17] Joshi A.Y., Iyer V.N., Hagan J.B., Sauver J.L.S., Boyce T.G. (2009). Incidence and Temporal Trends of Primary Immunodeficiency: A Population-Based Cohort Study. Mayo Clin. Proc..

[18] Boyle J.M., Buckley R.H. (2007). Population prevalence of diagnosed primary immunodeficiency diseases in the United States. J. Clin. Immunol..

[19] Moodley S., Goddard E., Levin M., Scott C., van Eyssen A., Davidson A., de Decker R., Wilmshurst J.M., Spitaels A., Eley B. (2020). A retrospective description of primary immunodeficiency diseases at Red Cross War Memorial Children’s Hospital, Cape Town, South Africa, 1975–2017. S. Afr. Med. J..

[20] Goda R., Sobh A., Nermeen G., Radwan N., Barbouche M.R., Ben-Mustapha I., Bousfiha A., Jeddane L., Mahlaoui N., Elfeky R. (2022). African Society for Immunodeciency (Asid) Guidelines for Diagnosis and Management of Inborn Errors of Immunity in Africa: Core Concept, Development and Initial Results. https://www.researchsquare.com/article/rs-2235434/v1.

[21] Aghamohammadi A., Rezaei N., Yazdani R., Delavari S., Kutukculer N., Topyildiz E., Ozen A., Baris S., Karakoc-Aydiner E., Kilic S.S. (2021). Consensus Middle East and North Africa Registry on Inborn Errors of Immunity. J. Clin. Immunol..

[22] Abolhassani H., Azizi G., Sharifi L., Yazdani R., Mohsenzadegan M., Delavari S., Sohani M., Shirmast P., Chavoshzadeh Z., Mahdaviani S.A. (2020). Global systematic review of primary immunodeficiency registries. Expert. Rev. Clin. Immunol..

[23] World Health Organization (2020). WHO Global TB Report.

[24] Boutayeb A. (2010). The Impact of Infectious Diseases on the Development of Africa. Handbook of Disease Burdens and Quality of Life Measures.

[25] Nkengasong J.N., Tessema S.K. (2020). Africa Needs a New Public Health Order to Tackle Infectious Disease Threats. Cell.

[26] Organization WHO (2022). Global Tuberculosis Report.

[27] Campbell M.C., Tishkoff S.A. (2008). African Genetic Diversity: Implications for Human Demographic History, Modern Human Origins, and Complex Disease Mapping. Annu. Rev. Genomics Hum. Genet..

[28] Petersen D.C., Steyl C., Scholtz D., Baker B., Abdullah I., Uren C., Möller M. (2022). African Genetic Representation in the Context of SARS-CoV-2 Infection and COVID-19 Severity. Front. Genet..

[29] Uren C., Möller M., van Helden P.D., Henn B.M., Hoal E.G. (2017). Population structure and infectious disease risk in southern Africa. Mol. Genet. Genomics.

[30] Wiener E.K., Buchanan J., Krause A., Lombard Z. (2023). Retrospective file review shows limited genetic services fail most patients—An argument for the implementation of exome sequencing as a first-tier test in resource-constrained settings. Orphanet J. Rare Dis..

[31] Bousfiha A.A., Jeddane L., El Hafidi N., Benajiba N., Rada N., El Bakkouri J., Kili A., Benmiloud S., Benhsaien I., Faiz I. (2014). First report on the Moroccan registry of primary immunodeficiencies: 15 Years of experience (1998–2012). J. Clin. Immunol..

[32] Cornelissen H.M., Glanzmann B., Van Coller A., Engelbrecht C., Abraham D.R., Reddy K., Möller M., Kinnear C., Glashoff R.H., Esser M. (2021). Mendelian susceptibility to mycobacterial disease in tuberculosis-hyperendemic South Africa. S. Afr. Med. J..

[33] Quinn J., Modell V., Orange J.S., Modell F. (2022). Growth in diagnosis and treatment of primary immunodeficiency within the global Jeffrey Modell Centers Network. Allergy Asthma Clin. Immunol..

[34] Leiding J.W., Holland S.M. (1993). Chronic Granulomatous Disease.

[35] Carranza C., Pedraza-Sanchez S., de Oyarzabal-Mendez E., Torres M. (2020). Diagnosis for Latent Tuberculosis Infection: New Alternatives. Front. Immunol..

[36] Menzies N.A., Swartwood N., Testa C., Malyuta Y., Hill A.N., Marks S.M., Cohen T., Salomon J.A. (2021). Time Since Infection and Risks of Future Disease for Individuals with Mycobacterium tuberculosis Infection in the United States. Epidemiology.

[37] Roya-Pabon C.L., Perez-Velez C.M. (2016). Tuberculosis exposure, infection and disease in children: A systematic diagnostic approach. Pneumonia.

[38] Abel L., Fellay J., Haas D.W., Schurr E., Srikrishna G., Urbanowski M., Chaturvedi N., Srinivasan S., Johnson D.H., Bishai W.R. (2018). Genetics of human susceptibility to active and latent tuberculosis: Present knowledge and future perspectives HHS Public Access. Lancet Infect. Dis..

[39] Ferrara G., Murray M., Winthrop K., Centis R., Sotgiu G., Migliori G.B., Maeurer M., Zumla A. (2012). Risk factors associated with pulmonary tuberculosis: Smoking, diabetes and anti-TNFα drugs. Curr. Opin. Pulm. Med..

[40] Seddon J.A., Chiang S.S., Esmail H., Coussens A.K. (2018). The Wonder Years: What Can Primary School Children Teach Us About Immunity to *Mycobacterium tuberculosis*?. Front. Immunol..

[41] Middelkoop K., Bekker L.-G., Myer L., Dawson R., Wood R. (2008). Rates of tuberculosis transmission to children and adolescents in a community with high adult HIV prevalence. Clin. Infect. Dis..

[42] Alcaïs A., Fieschi C., Abel L., Casanova J.-L. (2005). Tuberculosis in children and adults. J. Exp. Med..

[43] Snow K.J., Sismanidis C., Denholm J., Sawyer S.M., Graham S. (2018). The incidence of tuberculosis among adolescents and young adults: A global estimate. Eur. Respir. J..

[44] Upton C.M., Van Wijk R.C., Mockeliunas L., Simonsson U.S., McHarry K., van den Hoogen G., Muller C., von Delft A., van der Westhuizen H.M., Van Crevel R. (2022). Safety and efficacy of BCG re-vaccination in relation to COVID-19 morbidity in healthcare workers: A double-blind, randomised, controlled, phase 3 trial. EClinicalMedicine.

[45] Hesseling A.C., Caldwell J., Cotton M.F., Eley B.S., Jaspan H.B., Jennings K., Marais B.J., Nuttall J., Rabie H., Roux P. (2009). BCG vaccination in South African HIV-exposed infants—Risks and benefits. South Afr. Med. J..

[46] Clark M., Cameron D.W. (2006). The benefits and risks of bacille Calmette-Guérin vaccination among infants at high risk for both tuberculosis and severe combined immunodeficiency: Assessment by Markov model. BMC Pediatr..

[47] Marciano B.E., Huang C.Y., Joshi G., Rezaei N., Carvalho B.C., Allwood Z., Ikinciogullari A., Reda S.M., Gennery A., Thon V. (2014). BCG vaccination in patients with severe combined immunodeficiency: Complications, risks, and vaccination policies. J. Allergy Clin. Immunol..

[48] Bustamante J., Boisson-Dupuis S., Abel L., Casanova J.L. (2014). Mendelian susceptibility to mycobacterial disease: Genetic, immunological, and clinical features of inborn errors of IFN-γ immunity. Semin. Immunol..

[49] Adebamowo S.N., Francis V., Tambo E., Diallo S.H., Landouré G., Nembaware V., Dareng E., Muhamed B., Odutola M., Akeredolu T. (2018). Implementation of genomics research in Africa: Challenges and recommendations. Glob. Health Action..

[50] Mahdaviani S.A., Mansouri D., Jamee M., Zaki-Dizaji M., Aghdam K.R., Mortaz E., Khorasanizadeh M., Eskian M., Movahedi M., Ghaffaripour H. (2020). Mendelian Susceptibility to Mycobacterial Disease (MSMD): Clinical and Genetic Features of 32 Iranian Patients. J. Clin. Immunol..

[51] Xia L., Liu X.-H., Yuan Y., Lowrie D.B., Fan X.-Y., Li T., Hu Z.-D., Lu S.-H. (2022). An Updated Review on MSMD Research Globally and A Literature Review on the Molecular Findings, Clinical Manifestations, and Treatment Approaches in China. Front. Immunol..

[52] Bandari A.K., Muthusamy B., Bhat S., Govindaraj P., Rajagopalan P., Dalvi A., Shankar S., Raja R., Reddy K.S., Madkaikar M. (2019). A Novel Splice Site Mutation in IFNGR2 in Patients with Primary Immunodeficiency Exhibiting Susceptibility to Mycobacterial Diseases. Front. Immunol..

[53] Markle J.G., Martĺnez-Barricarte R., Ma C.S., Deenick E.K., Ramírez-Alejo N., Mele F., Latorre D., Mahdaviani S.A., Aytekin C., Mansouri D. (2018). Human IFN-γ immunity to mycobacteria is governed by both IL-12 and IL-23. Sci. Immunol..

[54] Rosain J., Kong X.-F., Martinez-Barricarte R., Oleaga-Quintas C., Ramirez-Alejo N., Markle J., Okada S., Boisson-Dupuis S., Casanova J., Bustamante J. (2019). Mendelian susceptibility to mycobacterial disease: 2014–2018 update. Immunol. Cell Biol..

[55] Casanova J.-L. (2015). Severe infectious diseases of childhood as monogenic inborn errors of immunity. Proc. Natl. Acad. Sci. USA.

[56] Amberger J.S., Bocchini C.A., Schiettecatte F., Scott A.F., Hamosh A. (2015). OMIM.org: Online Mendelian Inheritance in Man (OMIM^®^), an online catalog of human genes and genetic disorders. Nucleic Acids Res..

[57] Esteve-Solé A., Sologuren I., Martínez-Saavedra M.T., Deyà-Martínez A., Oleaga-Quintas C., Martinez-Barricarte R., Martin-Nalda A., Juan M., Casanova J.-L., Rodriguez-Gallego C. (2018). Laboratory evaluation of the IFN-γ circuit for the molecular diagnosis of Mendelian susceptibility to mycobacterial disease. Crit. Rev. Clin. Lab. Sci..

[58] Errami A., El Baghdadi J., Ailal F., Benhsaien I., Ouazahrou K., Abel L., Casanova J.-L., Boisson-Dupuis S., Bustamante J., Bousfiha A.A. (2023). Mendelian susceptibility to mycobacterial disease: An overview. Egypt. J. Med. Hum. Genet..

[59] Bousfiha A., Jeddane L., Picard C., Al-Herz W., Ailal F., Chatila T., Cunningham-Rundles C., Etzioni A., Franco J.L., Holland S.M. (2020). Human Inborn Errors of Immunity: 2019 Update of the IUIS Phenotypical Classification. J. Clin. Immunol..

[60] Errami A., Baghdadi J.E., Ailal F., Benhsaien I., Bakkouri J.E., Jeddane L., Rada N., Benajiba N., Mokhantar K., Ouazahrou K. (2022). Mendelian Susceptibility to Mycobacterial Disease (MSMD): Clinical, immunological and genetic features of 22 Patients from 15 Moroccan kindreds. J. Clin. Immunol..

[61] Angelidou A., Conti M.-G., Diray-Arce J., Benn C.S., Shann F., Netea M.G., Liu M., Potluri L.P., Sanchez-Schmitz G., Husson R. (2020). Licensed Bacille Calmette-Guérin (BCG) formulations differ markedly in bacterial viability, RNA content and innate immune activation. Vaccine.

[62] Mahomed H., Kibel M., Hawkridge T., Schaaf H.S., Hanekom W.A., Iloni K., Michaels D., Workman L., Verver S., Geiter L. (2006). The Impact of a Change in Bacille Calmette-Guérin Vaccine Policy on Tuberculosis Incidence in Children in Cape Town, South Africa. Pediatr. Infect. Dis. J..

[63] Jethwa H., Wong R., Abraham S. (2021). COVID-19 vaccine trials: Ethnic diversity and immunogenicity. Vaccine.

[64] Edwards K.M. (2015). Maternal antibodies and infant immune responses to vaccines. Vaccine.

[65] Cinicola B., Conti M.G., Terrin G., Sgrulletti M., Elfeky R., Carsetti R., Salinas A.F., Mortari E.P., Brindisi G., de Curtis M. (2021). The Protective Role of Maternal Immunization in Early Life. Front. Pediatr..

[66] Esser M.M., Potter P., Nortje R. (2016). Meeting the needs of primary immunodeficiency patients in South Africa—Some findings from the South African registry summary. Curr. Allergy Clin. Immunol..

[67] Kampmann B., Hemingway C., Stephens A., Davidson R., Goodsall A., Anderson S., Nicol M., Schölvinck E., Relman D., Waddell S. (2005). Acquired predisposition to mycobacterial disease due to autoantibodies to IFN-γ. J. Clin. Investig..

[68] Lye J.J., Williams A., Baralle D. (2019). Exploring the RNA Gap for Improving Diagnostic Yield in Primary Immunodeficiencies. Front. Genet..

[69] Meyts I., Bosch B., Bolze A., Boisson B., Itan Y., Belkadi A., Pedergnana V., Moens L., Picard C., Cobat A. (2016). Exome and genome sequencing for inborn errors of immunity. J. Allergy Clin. Immunol..

[70] Schwarze K., Buchanan J., Taylor J.C., Wordsworth S. (2018). Are whole-exome and whole-genome sequencing approaches cost-effective? A systematic review of the literature. Genet. Med..

[71] Zhang C., Hansen M.E.B., Tishkoff S.A. (2022). Advances in integrative African genomics. Trends Genet..

[72] Omotoso O.E., Teibo J.O., Atiba F.A., Oladimeji T., Adebesin A.O., Babalghith A.O. (2022). Bridging the genomic data gap in Africa: Implications for global disease burdens. Glob. Health.

[73] Pairo-Castineira E., Clohisey S., Klaric L., Bretherick A.D., Rawlik K., Pasko D., Walker S., Parkinson N., Fourman M.H., Russell C.D. (2021). Genetic mechanisms of critical illness in COVID-19. Nature.

[74] Swart Y., van Eeden G., Sparks A., Uren C., Möller M. (2020). Prospective avenues for human population genomics and disease mapping in southern Africa. Mol. Genet. Genomics.

[75] Gurdasani D., Carstensen T., Tekola-Ayele F., Pagani L., Tachmazidou I., Hatzikotoulas K., Karthikeyan S., Iles L., Pollard M.O., Choudhury A. (2015). The African Genome Variation Project shapes medical genetics in Africa. Nature.

[76] Tetikol H.S., Turgut D., Narci K., Budak G., Kalay O., Arslan E., Demirkaya-Budak S., Dolgoborodov A., Kabakci-Zorlu D., Semenyuk V. (2022). Pan-African genome demonstrates how population-specific genome graphs improve high-throughput sequencing data analysis. Nat. Commun..

[77] Adepoju P. (2022). Tackling Africa’s underrepresentation in genomics studies. Nat. Afr..

[78] Nica A.C., Dermitzakis E.T. (2013). Expression quantitative trait loci: Present and future. Philos. Trans. R. Soc. Lond. B Biol. Sci..

[79] Fairfax B.P., Humburg P., Makino S., Naranbhai V., Wong D., Lau E., Jostins L., Plant K., Andrews R., McGee C. (2014). Innate Immune Activity Conditions the Effect of Regulatory Variants upon Monocyte Gene Expression. Science.

[80] Piasecka B., Duffy D., Urrutia A., Quach H., Patin E., Posseme C., Bergstedt J., Charbit B., Rouilly V., MacPherson C.R. (2018). Distinctive roles of age, sex, and genetics in shaping transcriptional variation of human immune responses to microbial challenges. Proc. Natl. Acad. Sci. USA.

[81] Von Both U., Kaforou M., Levin M., Newton S.M. (2015). Understanding immune protection against tuberculosis using RNA expression profiling. Vaccine.

[82] Billington C., Toles O., Ebens C., Johnson A., Pozos T., Binstadt B., Albert F., Thielen B. (2023). Transcriptomic Approaches to Diagnosis of Inborn Errors of Immunity. Clin. Immunol..

[83] Bougarn S., Boughorbel S., Chaussabel D., Marr N. (2019). A curated transcriptome dataset collection to investigate inborn errors of immunity. F1000Research.

